# Autonomic function measurements for evaluating fatigue and quality of life in patients with breast cancer undergoing radiation therapy: a prospective longitudinal study

**DOI:** 10.1186/s13014-023-02362-w

**Published:** 2023-10-19

**Authors:** Miwa Aoki, Hirohiko Kuratsune, Sena Yamamoto, Toshiko Hirano, Kumiko Hanaeda, Yasuka Nishi, Tamami Okada, Maki Nadamura, Chiho Kobayashi, Emiko Sumita, Junko Gotou, Masahiko Koizumi, Harue Arao

**Affiliations:** 1 Department of Evidence-Based Clinical Nursing, Division of Health Sciences, Osaka University Graduate School of Medicine, 1-7 Yamadaoka, Suita, Osaka 565-0871 Japan; 2 Fatigue and Mental Health Check Center (FMCC), Co., Ltd., Osaka, Japan; 3 Department of Nursing, Osaka Red Cross Hospital, Osaka, Japan; 4 Department of Nursing, Wakayama Medical University Hospital, Wakayama, Japan; 5 Department of Nursing, Otemae Hospital, Osaka, Japan; 6 Department of Nursing, Kanazawa Medical University Hospital, Kahoku, Japan; 7 Department of Nursing, Omi Medical Center, Kusatsu, Japan; 8 Department of Nursing, Takatsuki Red Cross Hospital, Takatsuki, Japan; 9 Department of Nursing, Osaka Medical and Pharmaceutical University Hospital, Takatsuki, Japan; 10 Department of Medical Physics and Engineering, Division of Health Sciences, Osaka University Graduate School of Medicine, Suita, Japan

**Keywords:** Breast cancer, Fatigue, Autonomic nerve function, Radiation therapy

## Abstract

**Background:**

Fatigue during radiation therapy in women with breast cancer can decrease quality of life (QOL), yet it is often underestimated and needs to be evaluated objectively. This longitudinal study aimed to evaluate fatigue and QOL of women with breast cancer undergoing radiotherapy with a simple autonomic function measurement.

**Methods:**

Women with breast cancer who underwent postoperative radiotherapy in eight cancer care hospitals in Chubu and Kinki regions in Japan were recruited between October 2021 and June 2022. The women underwent a self-administered questionnaire that included the Cancer Fatigue Scale (CFS) and the Short Form-8 Health Survey (SF-8) and an autonomic nervous function measurement using a simple, non-invasive device before (T0, baseline), mid (T1), and at the end (T2) of treatment.

**Results:**

The 57 women showed similar trends, with CFS scores and log LF/HF ratio being the highest at T0 and significantly decreasing at T1 (both p < 0.05). The log LF/HF trends differed between those with high and low baseline log LF/HF values. Women with mental component summary (MCS) score improvement (T0 to T2) had the highest log LF/HF ratio at T0 and had significantly lower log LF/HF values at T1 and T2 than at T0 (p < 0.01 and p < 0.05, respectively). The change of (⊿) MCS from T0 to T1 was negatively correlated with ⊿log LF/HF from T0 to T1 (r = − 0.36, p < 0.01).

**Conclusions:**

Measurement of autonomic nerve function with a simple device is useful for objective fatigue assessment during radiotherapy. Psychological support is important as improvement in mental health helps improve autonomic nerve function and, in turn, fatigue.

**Supplementary Information:**

The online version contains supplementary material available at 10.1186/s13014-023-02362-w.

## Background

Breast cancer is the most common cancer among women worldwide, with new cases exceeding 2.2 million in 2020 [1]. Breast cancer can be effectively treated with early detection and multidisciplinary treatment, and the survival rate in breast cancer is as high as 90% in the highest countries [2]. Radiotherapy is provided to reduce the risk of postoperative recurrence and improve overall survival; however, it increases the acute side effects of fatigue. Fatigue is the most common side effect of radiotherapy; by the end of radiotherapy, 77–90% of patients with breast cancer experience fatigue [3–5]. Severe fatigue can lead to discontinuation of radiotherapy. Fatigue also continues after radiotherapy [4–6], and this long-term effect may interfere with activities of daily living [7–9]. Radiotherapy-related fatigue is also closely related to psychological aspects, with pretreatment anxiety, depressed mood, and sadness being predictors of fatigue [9–11]. It has also been reported that fatigue leads to a decrease in health-related quality of life (HRQoL) in the psychological dimensions as well as in the physical and social dimensions [12, 13].

The negative effects of fatigue may be attributed to characteristics of cancer-related fatigue, including radiotherapy-related fatigue, which is a subjective and multidimensional sense of the patient [14]. These multiple dimensions must be considered in the evaluation and management of fatigue. Several fatigue measurement scales that consider these characteristics have been developed [15, 16]. Nonetheless, because fatigue is subjective, physicians and nurses tend to underestimate patient fatigue [17]. Therefore, fatigue assessment using objective indicators and management that responds to biological reactions is crucial. One available indicator of cancer-related fatigue is autonomic nervous function. Both sympathetic and parasympathetic activities provide frequency-specific contributions to the heart rate [18]. Subsequent studies of R-R interval in populations with chronic stress have reported a decrease in normalized high-frequency (HF) component and an increase in normalized low-frequency (LF) component [19]. Since then, heart rate variability (HRV) with continuous heart rate monitoring has been used as a robust measure of autonomic function in chronic fatigue. In recent years, non-invasive and simple autonomic nervous function measurements have been developed as job stress screening or fatigue evaluation for Japanese employees [20, 21].

Cancer-induced fatigue is potentially associated with lower HRV in breast cancer survivors [22–24]. The mechanism of radiotherapy-related fatigue may indicate the usefulness of fatigue assessment using autonomic nervous function instruments. Proinflammatory cytokines are released owing to irradiation-induced tissue damage [25]. Activation of inflammatory cytokines and high levels of downstream biomarkers of cytokine activity are associated with radiotherapy-related fatigue [26, 27]. Given that inflammatory response is regulated by the autonomic nervous system [28, 29], the autonomic nervous function is thus an essential indicator of radiotherapy-related fatigue. However, no study has focused on the usefulness of autonomic function indicators as an objective measure of fatigue in patients with breast cancer undergoing radiotherapy. In addition, the existing measurement tools have limited clinical application because they are time-consuming, physically tedious, and costly. Visualization of fatigue using simple autonomic nervous function evaluation makes it possible to assess a patient's radiotherapy-related fatigue immediately. It leads to the appropriate intervention tailored to the patients’ fatigue status. Therefore, this study aimed to evaluate fatigue and QOL in patients with breast cancer undergoing radiotherapy by using simple autonomic function measurement. We hypothesized that autonomic function measurement would be useful in evaluating radiotherapy-related fatigue.

## Methods

### Study design and population

This multicenter, longitudinal study was conducted in eight designated cancer care hospitals in Chubu and Kinki regions in Japan between October 2021 and June 2022. The participants were women with newly diagnosed breast cancer who started whole breast radiotherapy or postmastectomy radiotherapy (PMRT) as adjuvant radiotherapy in the outpatient setting. The inclusion criteria were as follows: (a) females aged ≥ 20 years, (b) confirmed cancer diagnosis, (c) indicated for radiotherapy of 40–60 Gy, (d) able to complete the questionnaire and participate in the measurement of autonomic nervous function, and (d) obtained permission to participate in this study from a physician in breast surgery and radiology. The participants were recruited regardless of whether they received a sequential combination of chemotherapy and radiotherapy or a concurrent combination of hormone therapy and radiotherapy. The exclusion criteria were as follows: (a) bilateral breast and/or chest wall irradiation; (b) local recurrence, multiple primary cancer, and distant metastasis (Stage IV); (c) receiving palliative radiotherapy; (d) a history of cardiac disease or arrhythmia; (e) a history of mental disorders; and (f) failed to complete the survey within ± 1 day of the date established as the study point.

When planning the research protocol, the sample size to be assured at T2 was calculated at 56 by G*Power 3.1.9 with an effect size of 0.25, alpha error of 0.05, and power of 0.80, and we considered a 20% dropout rate. Due to a 25% dropout rate identified during the data collection process for T2, 75 participants were ultimately recruited.

### Study procedure and data collection

Nurses, as coresearchers, provided eligible patients with information about the study during their first radiology consultation. Patients who expressed interest in the study on the same day or at the time of the computed tomography (CT) scan for radiotherapy planning were educated about the study by the coresearcher. Their written informed consent was obtained. Data were collected using a self-administered questionnaire, and autonomic nervous function was measured during three time points as follows: (1) prior to radiotherapy at the time of the first consultation in the radiology department or CT scan for radiotherapy planning (T0: baseline), (2) mid-radiotherapy (i.e., day 7 or 8 of hypofractionation/day 12 or 13 of conventional fractionation (T1)), and (3) last day of radiotherapy (T2). For patients with boost irradiation, the third time point was the last day of conventional or hypofractionation therapy. The exposure dose for conventional fractionation was consistently at 50.00 Gy/25 Fr, while the dose for hypofractionation ranged from 40.55 Gy to 44.00 Gy/15–16 Fr. Hence, the data collection date for T1 was assigned based on calculations using the biologically effective dose: {n (fraction) × d (dose)} × {1 + d/ (α/β:10 as tumor cell)}. Only complete data on all three time points and the primary endpoints of subjective and objective fatigue were analyzed.

### Measures

#### Clinicodemographic characteristics

Demographic data (age, marital status, living arrangement, employment status, and time required for hospital visits) were obtained using a questionnaire. Clinical data (time since surgery, type of surgery, tumor stage, chemotherapy, time since chemotherapy, hormone therapy, the planned total dose of radiation, type or protocol of radiotherapy, Eastern Cooperative Oncology Group Performance Status, adverse events assessed using the Common Terminology Criteria for Adverse Events v 5.0, presence of comorbidity and mediations taken by participants) were obtained from medical records.

#### Subjective fatigue

Subjective fatigue was assessed with the Cancer Fatigue Scale (CFS) [15]. Briefly, the CFS is a 15-item scale that includes physical (7 items), affective (4 items), and cognitive (4 items) subscales. All items are rated on a 5-point Likert scale from 1 (not at all) to 5 (very much). The highest physical, affective, cognitive, and total CFS scores are 28, 16, 16, and 60, respectively: the higher the score, the higher the level of fatigue. The validity and reliability of this scale were confirmed in a development study [15]. In the present study, Cronbach’s alpha for the physical subscale was 0.83; affective subscale, 0.87; cognitive subscale, 0.72; and total CFS, 0.75.

#### Objective fatigue

Objective fatigue was evaluated according to autonomic nervous function measured using the vital monitor VM600 system (Fatigue Science Laboratory Inc, Osaka, Japan). The VM600 system is a non-invasive, simple healthcare device attached to the user’s fingertip. It can measure variations in the R–R interval from electrocardiography and in the A–A interval from photoplethysmography. In this study, measures of autonomic nervous function were obtained via frequency analysis of the variation of the heartbeat interval (R–R interval) for 2 min [20]. Participants were briefed about the procedure, and then the VM600 was attached. The participant sat quietly with their eyes closed for 2 min in a private room or similar place. The autonomic nervous function was assessed using the following indicators: high frequency (HF): 0.15–0.40 Hz, which primarily reflects parasympathetic nerve modulation [30]; low frequency (LF): 0.04–0.15 Hz, which is primarily regulated by sympathetic nerve system [30, 31]; and LF/HF ratio, which indicates the balance ratio of autonomic nervous function [31].

#### Health-related quality of life

We used the Short Form-8 Health Survey (SF-8) acute version, abbreviated Japanese version to assess HRQoL in the past week [32]. The SF-8 comprises eight subscales: physical functioning, bodily pain, role physical, general health, vitality, social functioning, role emotional, and mental health. Each item is rated on a 5- or 6-point Likert scale. The physical component summary (PCS) and mental component summary (MCS) are also calculated based on the eight subscales. Scoring is norm-based with national standard values. A score of 50 is the national average for Japanese, with higher scores indicating a better QOL. The reliability coefficients of parallel forms were reported to be 0.90 for PCS and 0.85 for MCS in the English version, and the validity was also confirmed in the Japanese version [32].

### Statistical analysis

Descriptive statistics summarized the participant characteristics. The mean scores of variables were calculated at all the time points. For autonomic nervous function, measured values were log-transformed to normalize them. The normal distributions of autonomic nervous function and CFS were confirmed by performing the Q–Q plot and Shapiro–Wilk test. Variables were compared among the time points using repeated measures analysis of variance (ANOVA) with the Bonferroni method for post-hoc analysis (if significant). Subsequently, the participants were classified into two groups based on two patterns: (i) the amount of change (⊿) from T0 to T2 in the MCS of SF-8 and (ii) the level of log LF/HF at T0. The participants with log LF/HF values of < 0.301 and ≥ 0.301 at T0 were classified into the normal log LF/HF group and the high log LF/HF group, respectively. This was because an LF/HF < 2 (log LF/HF < 0.301) was considered a balanced ratio similar to resting conditions in daily life [33]. Repeated-measures ANOVA and the Bonferroni method for post-hoc analysis were used for comparison of autonomic nervous function and the MCS and PCS of the SF-8 between the time points. Student t-tests were used to compare the two groups’ autonomic nervous function and the MCS and PCS of the SF-8. Chi-square tests or Fisher’s exact tests were used to compare participant characteristics between the groups. In addition, the association of ⊿MCS with ⊿log LF/HF was examined using Pearson’s correlation. All statistical analyses were performed using SPSS Ver. 27 (IBM Japan Ltd., Tokyo, Japan). A two-tailed p-value of 0.05 was considered statistically significant.

## Results

### Participant characteristics

**Fig. 1:**
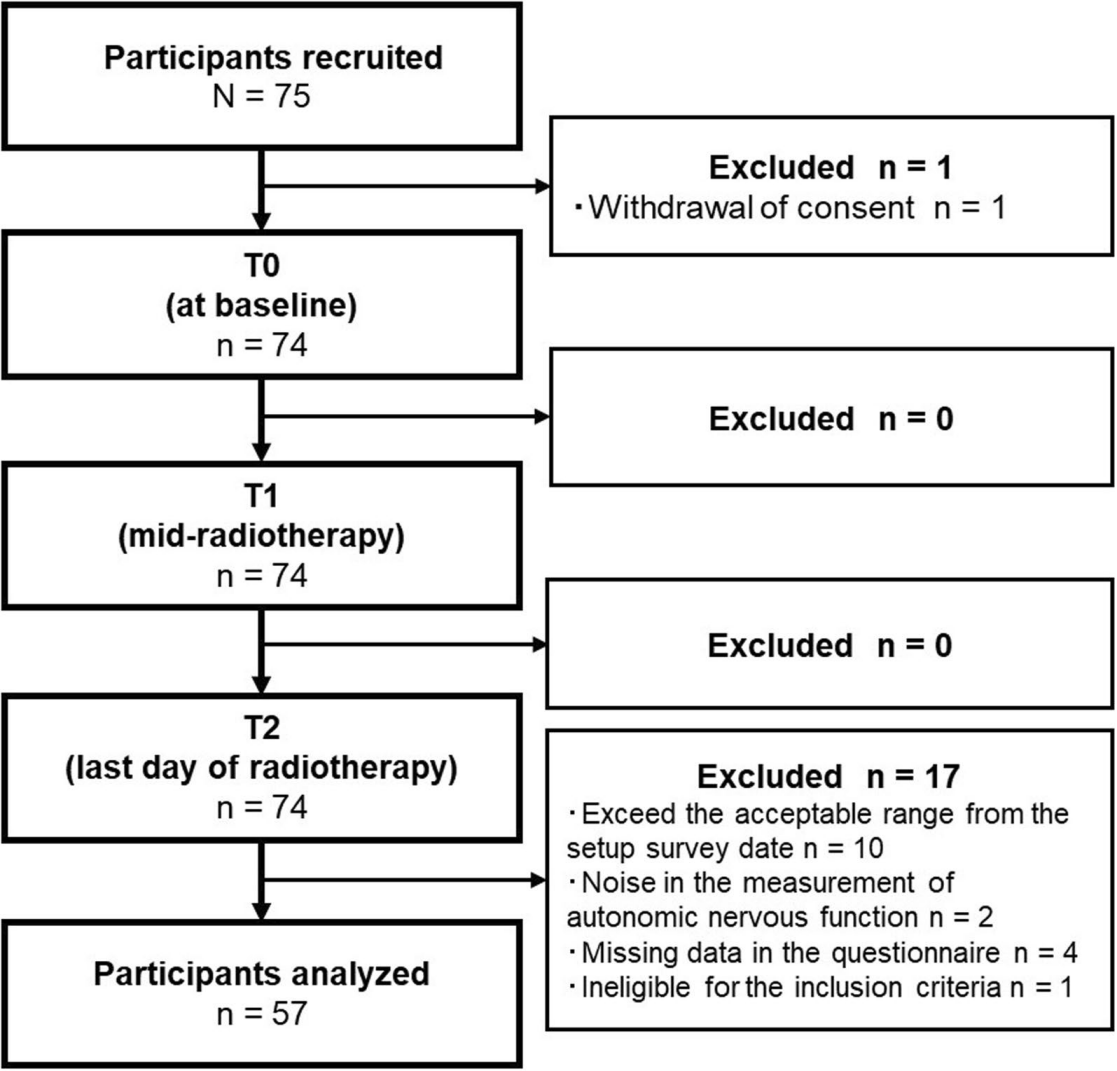
Participant enrollment flowchart

The participant enrollment flowchart is shown in Fig. 1, with 57 participants included in the final analysis. The participants' mean age was 54.86 years, and the median number of days elapsed since surgery was 45.5 days (Table 1). There were 24 (42.1%) and 19 (33.3%) participants with stage I and II diseases, respectively. Whole breast irradiation after breast-conserving surgery was performed in 44 (77.2%) participants, while hypofractionated radiotherapy was performed in 36 (63.2%) participants. Overall, 23 (40.4%) participants received chemotherapy before radiotherapy, and 27 (47.4%) participants received combined hormone therapy and radiotherapy. The participants had underlying diseases, including hypertension in eight patients (14.0%), asthma in five patients (8.8%), dyslipidemia in three patients (5.3%), and diabetes mellitus in two patients (3.5%). Of these patients, three (5.3%) were taking Ca^2+^ channel α2δ ligand, three (5.3%) were taking HMG-CoA reductase inhibitors, and one (1.8%) each was taking an opioid analgesic, nonopioid analgesic, benzodiazepine, orexin receptor antagonist, and others.

**Table 1:** Participant characteristics

Variable	n	%	Mean ± SD	Median	Range
Age, years			54.86 ± 10.45		34–78
Time since surgery, days				45.5	19–280
Tumor stage					
Stage 0	7	12.3			
Stage I	24	42.1			
Stage II	19	33.3			
Stage II	4	7.0			
Type of surgery					
Breast conserving surgery	44	77.2			
Mastectomy	13	22.8			
Chemotherapy					
Neo adjuvant	9	15.8			
Adjuvant	13	22.8			
Both neoadjuvant and adjuvant	1	1.8			
No	34	59.6			
Time since chemotherapy, days				39	13–94
Hormone therapy					
Antiestrogens (Tamoxifen)	16	28.1			
Aromatase inhibitors	11	19.3			
No	30	52.6			
Planned for a radiation total dose, Gy				50	40–60
Type of radiotherapy					
After breast-conserving surgery radiotherapy	44	77.2			
Postmastectomy radiotherapy (PMRT)	13	22.8			
Protocol of radiotherapy					
Hypofractionated	36	63.2			
Conventionally fractionated	21	36.8			
Marital status					
Unmarried	13	22.8			
Married	41	71.9			
Other	3	5.3			
Time required for hospital visit					
Less than 30 min	28	49.1			
30 min or more	24	42.1			
Living arrangement					
Living with family	49	86.0			
Alone	8	14.0			
Employment status					
Working	37	64.9			
Unemployed	20	35.1			
Presence of comorbidity					
Yes	38	66.7			
No	19	33.3			
Type of comorbidity^a^					
Hypertension	8	14.0			
Uterine fibroids	6	10.5			
Asthma	5	8.8			
Gallstone	3	5.3			
Dyslipidemia	3	5.3			
Diabetes mellitus	2	3.5			
Appendicitis	2	3.5			
Ovarian cystoma	2	3.5			
Osteoporosis	2	3.5			
Allergic rhinitis	2	3.5			
ECOG PS					
0	56	98.2			
1	1	1.8			
Symptoms (baseline)^b^					
Pain					
Grade1	6	10.5			
Lymphedema					
Grade1	1	1.8			
Grade2	1	1.8			
Pruritus					
Grade1	1	1.8			
Hypersomnia					
Grade1	1	1.8			

The values of some variables do not add up to 100% owing to missing values

ECOG PS, Eastern Cooperative Oncology Group Performance Status; SD, standard deviation

a Multiple responses, only diseases for which more than one person responded are selected

b Common Terminology Criteria for Adverse Events (CTCAE) v5.0

The cumulative irradiation dose in T1 was 20.20 ± 1.50 Gy (range, 15.96–22.50 Gy) for hypofractionated radiotherapy and 25.24 ± 1.18 Gy (range, 24.00–28.00 Gy) for conventionally fractionated radiotherapy. In T2, it was 41.82 ± 1.24 Gy (range, 39.90–44.00 Gy) for hypofractionated radiotherapy and 50.00 Gy for conventionally fractionated radiotherapy.

### Changes in subjective fatigue and HRQoL

**Fig. 2:**
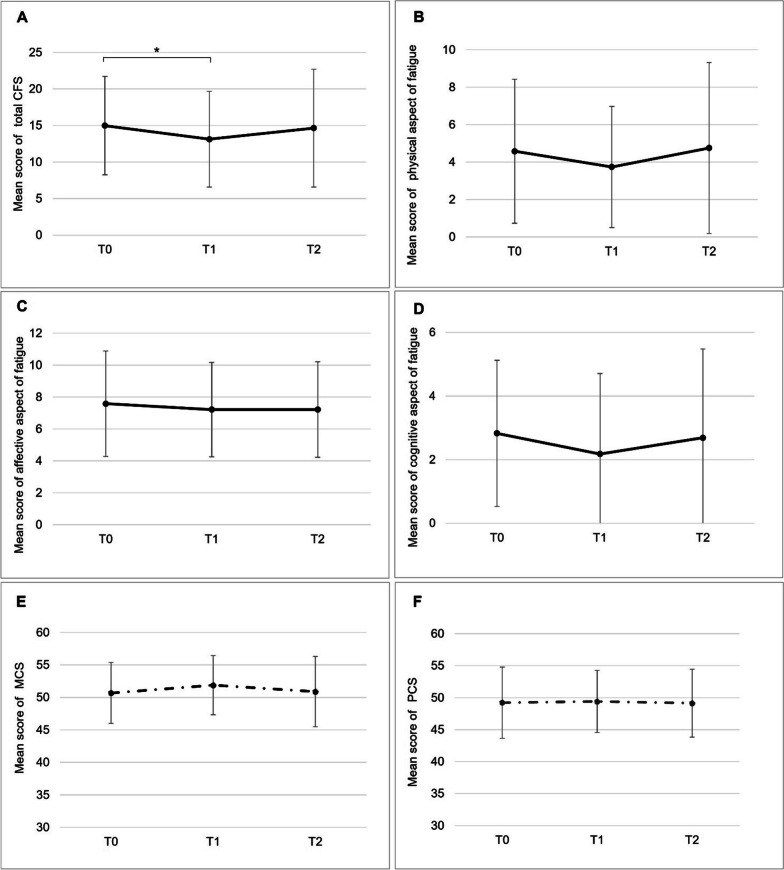
Changes in the mean scores of the Cancer Fatigue Scale (CFS) and the Short Form-8 Health Survey (SF-8) over time. **A** Total scale score of CFS. **B** Score of physical aspect of fatigue. **C** Score of affective aspect of fatigue. **D** Score of cognitive aspect of fatigue. **E** MCS score. **F** PCS score. For Bonferroni analysis, *p < 0.05. MCS, mental component summary; PCS, physical component summary

The CFS score changed over time, as shown in Fig. 2A–D. The mean CFS score was the highest at T0, decreased significantly at T1, and then increased at T2 to the same level as T0 (T0 vs. T1: 14.98 vs. 13.12, p = 0.02, T0 vs. T2: 14.98 vs. 14.65, p = 1.00). Physical fatigue was 4.58 at T0, decreased to 3.74 at T1, and then increased to 4.75 at T2, but the difference was insignificant (p = 0.11). Cognitive fatigue showed the same trend as physical fatigue, with a significant difference overall (p = 0.04) but not between each study point (T0 vs. T1: 2.82 vs. 2.18, p = 0.06, T0 vs. T2: 2.82 vs. 2.68, p = 1.00). Affective fatigue was also not significantly different from the 7-point range among the time points (p = 0.49). The SF-8 MCS scores were the lowest (50.69) at T0, increased to 51.88 at T1, and then decreased to 50.89 at T2. However, the differences among the time points were insignificant (p = 0.13; Fig. 2E). The PCS scores at all time points remained in the 49-point range, with no significant differences (p = 0.91; Fig. 2F).

### Changes in objective fatigue

**Fig. 3:**
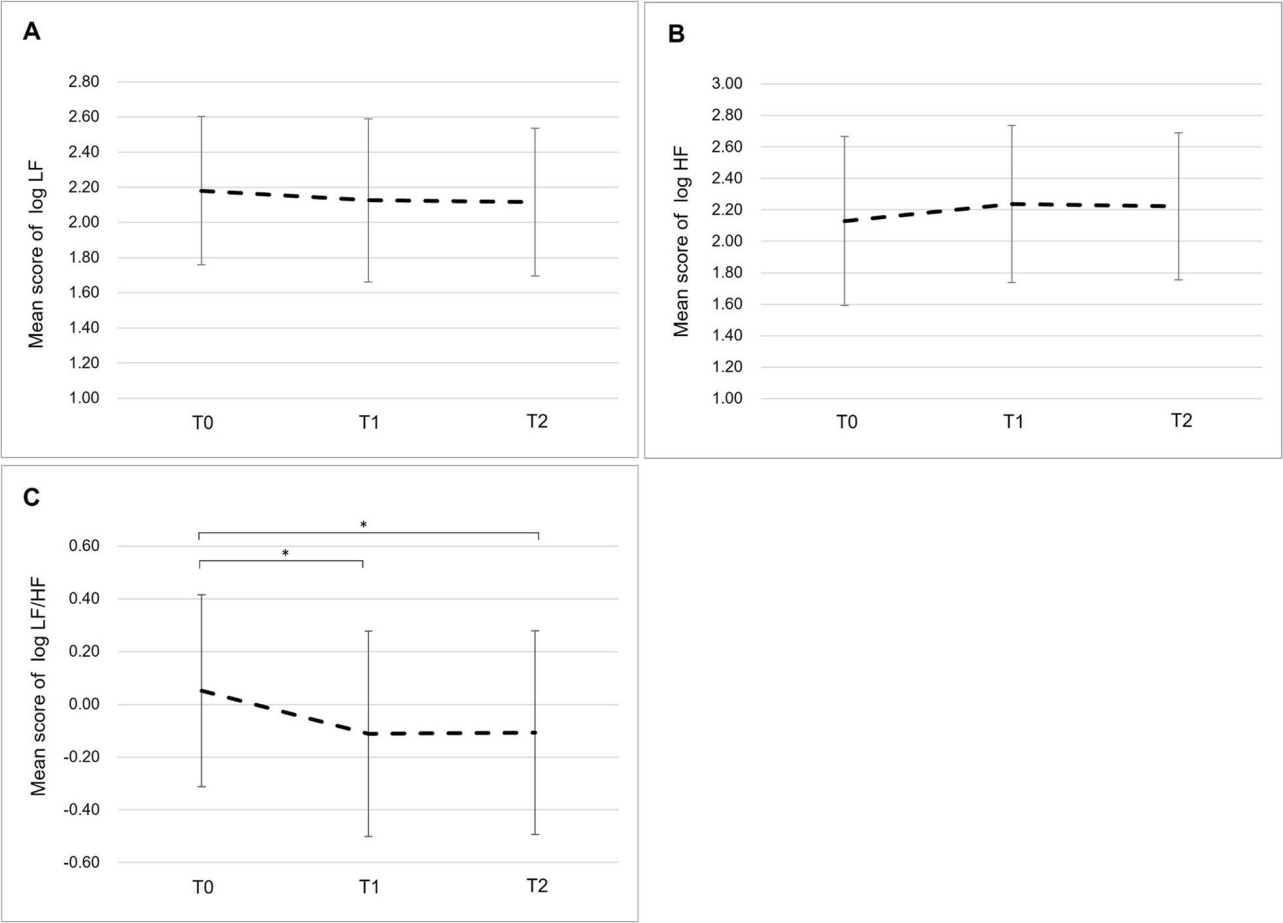
Changes in autonomic nervous function over time. **A** The mean score of log LF. **B** The mean score of log HF. **C** The mean log LF/HF ratio. For Bonferroni analysis **A–C**, *p < 0.05. LF/HF, low frequency/high frequency; HF, high frequency; LF, low frequency

The changes in autonomic nervous function are shown in Fig. 3. The mean log LF/HF value at T0 was 0.05, and the values were significantly lower at T1 and T2 than at T0 (T1: − 0.11, p = 0.03; T2: − 0.11, p = 0.04), but differences in log HF and log LF were not significant (p = 0.16 and p = 0.50, respectively). The mean log HF increased slightly from 2.13 at T0 to 2.24 at T1 and 2.22 at T2 (p = 0.16). Meanwhile, log LF decreased slightly from 2.18 at T0 to 2.13 at T1 and 2.12 at T2 (p = 0.50).

### Changes in autonomic nervous function according to MCS improvement

**Fig. 4:**
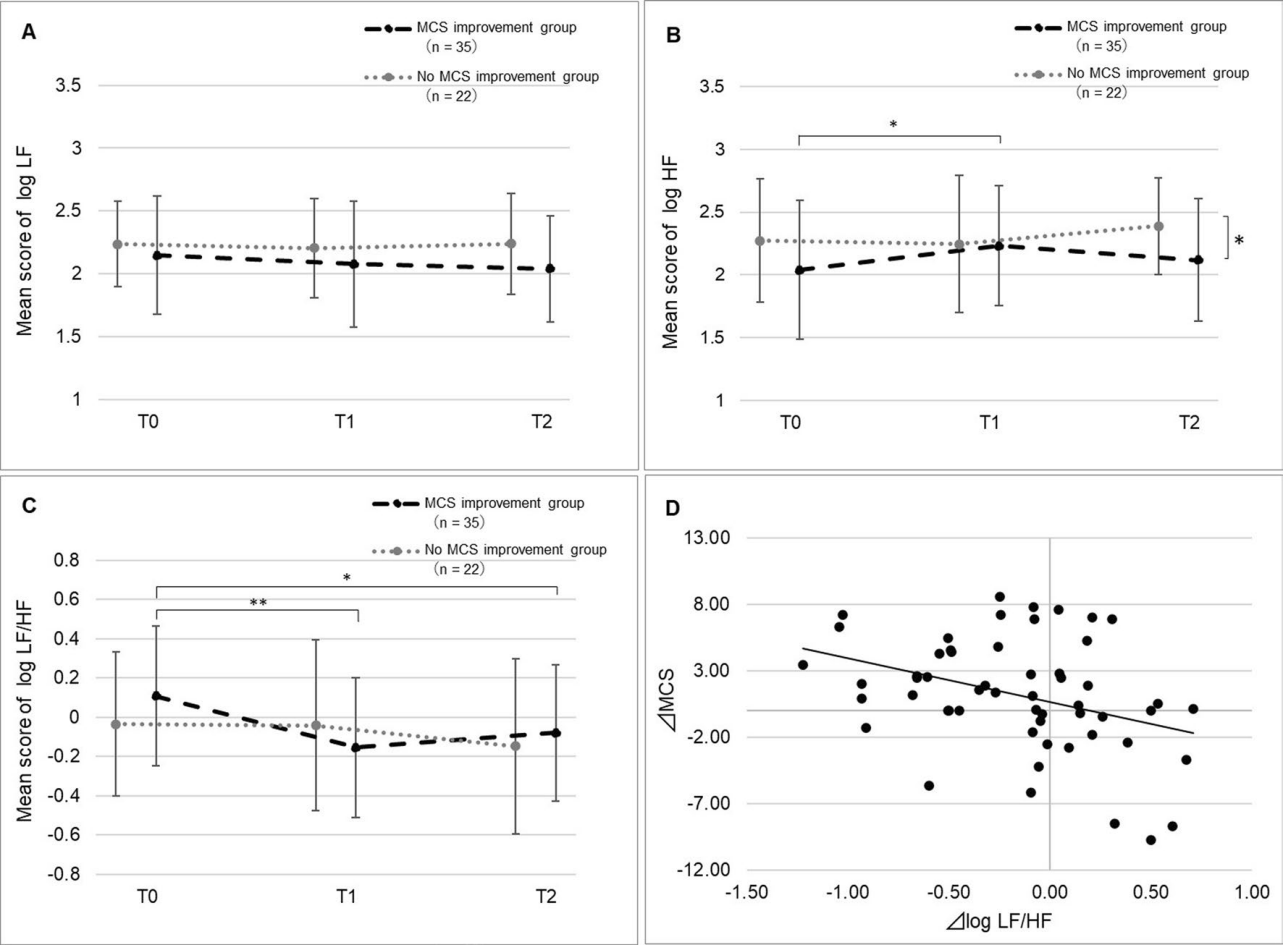
Changes in autonomic nervous function according to MCS improvement. **A** The mean score of log LF. **B** The mean score of log HF. **C** The mean score of the log LF/HF ratio. **D** Correlation between ⊿MCS from T0 to T1 and ⊿log LF/HF from T0 to T1; ⊿, the amount of change; T0 to T1, between prior to radiotherapy (T0, baseline) and mid-radiotherapy (T1). ⊿MCS of T0 to T2 > 0, the MCS improvement group; ⊿MCS of T0 to T2 ≤ 0, the no MCS improvement group; T0 to T2, between prior to radiotherapy (T0, baseline) and last day of radiotherapy (T2). Comparison between time points: for Bonferroni analysis (**A**, **B**, **C**), *p < 0.05, ** *p* < 0.01. Comparison between groups: for t-test (**A**, **B**, **C**), * *p* < 0.05. For Pearson’s coefficient (**D**). LF/HF, low frequency/high frequency; HF, high frequency; LF, low frequency; MCS, mental component summary

Autonomic nervous function in the participants with and without MCS improvement is shown in Fig. 4A–C. In the MCS improvement group, the log LF/HF ratio was the highest at T0 and significantly decreased at T1 and T2 (T0 vs. T1: 0.11 vs. − 0.15, p = 0.002; T0 vs. T2: 0.11 vs. − 0.08, p = 0.03). In addition, log HF in the MCS improvement group significantly increased from 2.04 at T0 to 2.23 at T1 (p = 0.02). In the no MCS improvement group, there were no significant differences in log LF/HF, log HF, and log LF across T0, T1, and T2 (p = 0.52, p = 0.39, and p = 0.93, respectively). The participant characteristics did not differ significantly among those with and without MCS improvement (Additional file 1: Appendix 1).⊿MCS from T0 to T1 showed a significantly negative correlation to ⊿log LF/HF from T0 to T1 (r = − 0.36, p = 0.007) (Fig. 4D).

### Changes in autonomic nervous function and HRQoL according to the baseline log LF/HF ratio

**Fig. 5:**
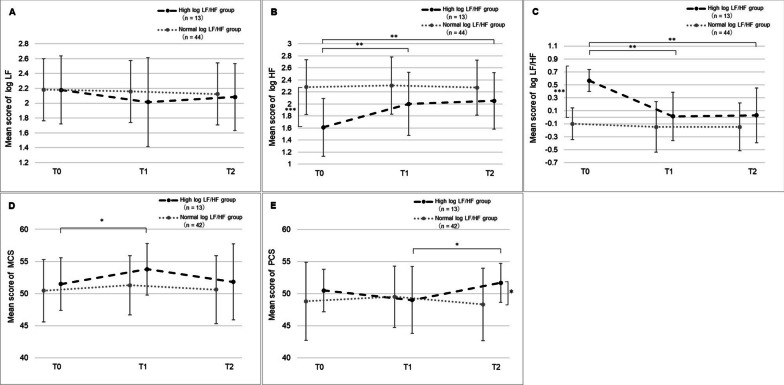
Changes in autonomic nervous function according to the baseline log LF/HF ratio. **A** The mean score of log LF. **B** The mean score of log HF. **C** The mean score of the log LF/HF ratio. **D** The mean score of MCS. **E** The mean score of PCS. Log LF/HF ≥ 0.301, the high log LF/HF group; log LF/HF < 0.301, the normal log LF/HF group. Comparison between time points: for Bonferroni analysis, *p < 0.05, ** *p* < 0.01. Comparison between groups: for t-test, *p < 0.05, ***p < 0.001. Owing to missing values, **A–E** have different n numbers in the normal log LF/HF group. LF/HF, low frequency/high frequency; HF, high frequency; LF, low frequency; MCS, mental component summary; PCS, physical component summary

Autonomic nervous function over time according to the log LF/HF ratio at T0 is shown in Fig. 5A–C. In the high baseline log LF/HF group, log LF/HF and log HF at T1 and T2 significantly differed from that at T0. Log LF/HF was significantly lower at T1 and T2 (T0 vs. T1: 0.57 vs. 0.01, p = 0.002, T0 vs. T2: 0.57 vs. 0.03, p = 0.004), and log HF was significantly higher at T1 and T2 (T0 vs. T1: 1.61 vs. 2.00, p = 0.003, T0 vs. T2: 1.61 vs. 2.05, p = 0.005). Log LF did not differ significantly among the time points (p = 0.44). Meanwhile, in the normal log LF/HF group, there were no significant differences in log LF/HF, log HF, and log LF across all the time points (p = 0.70, p = 0.88, and p = 0.70, respectively). The participants with high log LF/HF also showed significant changes in HRQoL over time, while those with normal log LF/HF showed no significant changes (Fig. 5D–E). The MCS scores in the high log LF/HF group significantly increased from 51.48 at T0 to 53.79 at T1 (p = 0.02). Similarly, the PCS scores in the high log LF/HF group significantly increased from 49.02 at T1 to 51.70 at T2 (p = 0.03). There were no significant differences in participant characteristics between the two groups (Additional file 2: Appendix 2).

## Discussion

This study found that fatigue levels were the highest among patients with breast cancer undergoing radiotherapy. The CFS scores for subjective fatigue and log LF/HF showed similar trends, peaking at baseline (T0) and significantly decreasing during mid-radiotherapy (T1). To our knowledge, this is the first study to evaluate fatigue and QOL using a convenient autonomic function measuring device in patients with breast cancer undergoing radiotherapy. The findings might indicate that objective evaluation of fatigue according to autonomic function assessment is beneficial and provide an important basis for developing appropriate interventions to reduce fatigue.

Our result showed similar changes over time in both CFS and log LF/HF, and the opposite trend of these changes in log HF, indicating the possibility of evaluating the pathophysiology of fatigue by measuring autonomic nervous function. Our results supported the positive correlation between LF/HF and fatigue [34]. The mechanisms for those with fatigue have increased sympathetic activity and decreased parasympathetic activity, resulting in an imbalance of the autonomic nervous system [35] in the chronic fatigue area. The present results may provide evidence that log LF/HF can be used to evaluate radiotherapy-related fatigue. A study of patients with lung cancer with targeted therapy or chemotherapy have reported an association between fatigue and LF/HF [36]. However, post-treatment of survivors of breast cancer revealed an association between HRV and fatigue, focusing on only parasympathetically-mediated HRV, including HF [22, 24]. Our results could provide new insights into the mechanism of radiotherapy-related fatigue for patients with breast cancer.

Importantly, we found that changes in autonomic nervous function over time can be evaluated according to MCS scores. In the MCS improvement group, log LF/HF was significantly lower; conversely, log HF was significantly higher at T1 than at T0. A significant negative correlation was observed between ⊿MCS and ⊿log LF/HF from T0 to T1. Prior research suggested that those with lower psychological well-being had higher LF/HF scores [37], and HF is associated with mental health-related indicators such as anxiety and depression [38]. Our results were consistent with these findings. Therefore, the mechanism of improved log LF/HF in the MCS improvement group could be assumed to stem from increasing log HF that occurs with stress relief. Similar mechanisms were found for fatigue. Our findings might suggest that improved mental HRQoL led to better autonomic function balance. Maintaining the mental HRQoL during the treatment may improve autonomic nervous function and fatigue for patients with breast cancer undergoing radiotherapy.

Notably, fatigue scores are the highest at baseline in the current study. This contrasts with several reports that fatigue scores peaked at the end of radiotherapy [5, 6, 39]. When the participants were divided into two groups according to the log LF/HF at baseline, the high log LF/HF group showed decreased log LF/HF and increased log HF during and at the end of treatment. These results contradict previous data suggesting that fatigue during treatment was more intense in patients with fatigue before treatment [10, 40]. These authors described fatigue in relation to anxiety states. In the current study, low mental HRQoL before treatment may result in high log LF/HF. Patients with higher log LF/HF ratios of pretreatment are presumed to have lower mental health function and should be considered for pretreatment interventions for fatigue. Thus, fatigue and mental health should be appropriately assessed from the pretreatment using autonomic function measurement. However, our study did not identify differences in participant characteristics according to the level of log LF/HF at baseline (see Additional file 2: Appendix 2), nor did it adequately focus on anxiety and other factors related to mental HRQoL. In addition, several outbreaks of COVID-19 occurred in Japan during the study period, which may have affected the patients' mental health condition before the start of radiotherapy. Future research should focus on factors associated with fatigue and autonomic function and detailed psychological variables.

## Implications for practice

Psychological support is essential to reduce radiotherapy-related fatigue. We demonstrated that the measurement of autonomic nervous function with a simple, non-invasive device helps assess fatigue before radiotherapy for breast cancer. Autonomic nervous function and subjective fatigue at baseline should be assessed throughout radiotherapy to identify targets for fatigue interventions. Measurement results should be shared with the patients, and they should be assisted in arranging their activities of daily living and social activities according to the degree of fatigue. The patient’s mental health should also be assessed at the beginning of treatment to evaluate the causes of fatigue and/or the effects associated with fatigue. In addition, for patients with breast cancer undergoing radiotherapy, a better mental HRQoL is beneficial for maintaining or improving the balance of autonomic nervous function and avoiding fatigue.

## Limitations

The present study has some limitations. First, the conditions during the measurement of autonomic function could not be standardized and controlled across all the outpatient radiology departments of the study sites. The autonomic nervous function is affected by circadian rhythms [41]. However, we could not measure autonomic nervous function under specific and similar conditions as the participants’ personal and treatment schedules were prioritized to reduce the burden. Next, types of comorbidity and medications taken by participants are too diverse and individualized to examine relationships among those factors, autonomic nervous function, and fatigue. The clinical laboratory data of the participants were also not collected in this study. Future studies require a study design that takes into account clinical laboratory data (e.g., morphology, electrolytes, and inflammatory processes), pharmacotherapy (e.g., opioid analgesics, adjuvant analgesics, and antipsychotics), and comorbidity, which might relate to fatigue of patients with breast cancer. Third, there may be selection bias because our study was conducted at designated cancer care hospitals in Japan. Lastly, this study was conducted during the COVID-19 pandemic, and changes in the treatment environment and the participants’ psychological distress owing to the pandemic may have potentially affected the results of autonomic nervous function and mental HRQoL assessments.

## Conclusions

Fatigue can be objectively assessed according to autonomic nerve function evaluated with a non-invasive and specificdevice among patients with breast cancer undergoing radiotherapy. Better mental HRQoL positively contributes to the balance of autonomic nervous function. Patients undergoing radiotherapy need psychological care before and during radiotherapy to manage fatigue. The log LF/HF ratio at baseline differs between the high log LF/HF group and the normal log LF/HF group. Thus, changes in autonomic nervous function over time, along with subjective fatigue prior to radiotherapy, should be evaluated to identify fatigue care targets.

### Supplementary Information

**Additional file 1:** **Appendix 1.** Comparison of participant characteristics according to with and without MCS improvement of T0 to T2. ^†^χ^2^ test, ^‡^Fisher's exact test. ⊿MCS of T0 to T2 > 0, the MCS improvement group; ⊿MCS of T0 to T2 ≤ 0, the no MCS improvement group; ⊿, the amount of change; T0 to T2, between prior to radiotherapy (T0, baseline) and last day of radiotherapy (T2). Abbreviations: MCS: mental component summary.

**Additional file 2:** **Appendix 2.** Comparison of participant characteristics according to the level of log LF/HF at baseline. ^†^χ^2^ test, ^‡^Fisher's exact test. Log LF/HF ≥ 0.301, the high log LF/HF group; log LF/HF < 0.301, the normal log LF/HF group. Abbreviations: LF/HF, low frequency/high frequency.

## Data Availability

The datasets used and/or analyzed during the current study are available from the corresponding author upon reasonable request.

## Abbreviations

HRQoL: Health-related quality of life
HF: High frequency
LF: Low frequency
HRV: Heart rate variability
PMRT: Postmastectomy radiotherapy
CT: Computed tomography
CFS: Cancer Fatigue Scale
PCS: Physical component summary
MCS: Mental component summary
ANOVA: Analysis of variance
