# Supplementary material for: Autonomic function measurements for evaluating fatigue and quality of life in patients with breast cancer undergoing radiation therapy: a prospective longitudinal study
Source: Radiat Oncol. 2023 Oct 19;18:171. doi: 10.1186/s13014-023-02362-w (PMC10585884; doi:10.1186/s13014-023-02362-w)
Supplement: Supplementary file 1 — Additional file 1: Appendix 1. Comparison of participant characteristics according to with and without MCS improvement of T0 to T2. †χ2 test, ‡Fisher's exact test. ⊿MCS of T0 to T2 > 0, the MCS improvement group; ⊿MCS of T0 to T2 ≤ 0, the no MCS improvement group; ⊿, the amount of change; T0 to T2, between prior to radiotherapy (T0, baseline) and last day of radiotherapy (T2). Abbreviations: MCS: mental component summary. [file 13014_2023_2362_MOESM1_ESM.docx]

**Appendix 1.** Comparison of participant characteristics according to with and without MCS improvement of T0 to T2.

|  | | MCS | | *p* |
| --- | --- | --- | --- | --- |
|  |  | No improvement  group  n (%) | Improvement group  n (%) |  |
| Age | Younger than 54 years | 11 (40.7) | 16 (59.3) | 0.75^†^ |
|  | 54 year or older | 11 (36.7) | 19 (63.3) |  |
| Tumor stage | Stage 0,1 | 12 (38.7) | 19 (61.3) | 0.77^†^ |
|  | Stage 2,3 | 8 (34.8) | 15 (65.2) |  |
| Type of surgery | Breast conserving surgery | 17 (38.6) | 27 (61.4) | 0.99^†^ |
|  | Mastectomy | 5 (38.5) | 8 (61.5) |  |
| Time since surgery | Less than 45 days | 10 (40.0) | 15 (60.0) | 1.00^†^ |
|  | 45 days or more | 12 (40.0) | 18 (60.0) |  |
| Chemotherapy | Yes | 9 (39.1) | 14 (60.9) | 0.95^†^ |
|  | No | 13 (38.2) | 21 (61.8) |  |
| Hormone therapy | Antiestrogens (Tamoxifen) | 8 (44.4) | 10 (55.6) | 0.69^†^ |
|  | Aromatase inhibitors | 4 (44.4) | 5 (55.6) |  |
|  | No | 10 (33.3) | 20 (66.7) |  |
| Protocol of radiotherapy | Hypofractionated | 15 (41.7) | 21 (58.3) | 0.53^†^ |
|  | Conventionally fractionated | 7 (33.3) | 14 (66.7) |  |
| Comorbidity | Yes | 14 (36.8) | 24 (63.2) | 0.70^†^ |
|  | No | 8 (42.1) | 11 (57.9) |  |
| Time required for hospital visit | Less than 30 minutes | 9 (32.1) | 19 (67.9) | 0.31^†^ |
|  | 30 minutes or more | 11 (45.8) | 13 (54.2) |  |
| Marital status | Married | 15 (36.6) | 26 (63.4) | 0.62^†^ |
|  | Unmarried or other | 7 (43.8) | 9 (56.3) |  |
| Living arrangement | Living with family | 19 (38.8) | 30 (61.2) | 1.00^‡^ |
|  | Alone | 3 (37.5) | 5 (62.5) |  |
| Employment status | Working | 16 (43.2) | 21 (56.8) | 0.33^†^ |
|  | Unemployed | 6 (30.0) | 14 (70.0) |  |

^†^χ^2^ test, ^‡^Fisher's exact test

⊿MCS of T0 to T2 > 0, the MCS improvement group; ⊿MCS of T0 to T2 ≤ 0, the no MCS improvement group; ⊿, the amount of change; T0 to T2, between prior to radiotherapy (T0, baseline) and last day of radiotherapy (T2).

Abbreviations: MCS: mental component summary
