# Supplementary material for: Autonomic function measurements for evaluating fatigue and quality of life in patients with breast cancer undergoing radiation therapy: a prospective longitudinal study
Source: Radiat Oncol. 2023 Oct 19;18:171. doi: 10.1186/s13014-023-02362-w (PMC10585884; doi:10.1186/s13014-023-02362-w)
Supplement: Supplementary file 2 — Additional file 2: Appendix 2. Comparison of participant characteristics according to the level of log LF/HF at baseline. †χ2 test, ‡Fisher's exact test. Log LF/HF ≥ 0.301, the high log LF/HF group; log LF/HF < 0.301, the normal log LF/HF group. Abbreviations: LF/HF, low frequency/high frequency. [file 13014_2023_2362_MOESM2_ESM.docx]

**Appendix 2.** Comparison of participant characteristics according to the level of log LF/HF at baseline.

|  | | Log LF/HF | | *p* |
| --- | --- | --- | --- | --- |
|  |  | Normal group  n (%) | High group  n (%) |  |
| Age | Younger than 54 years | 23 (85.2) | 4 (14.8) | 0.17^†^ |
|  | 54 year or older | 21 (70.0) | 9 (30.0) |  |
| Tumor stage | Stage 0,1 | 24 (77.4) | 7 (22.6) | 0.77^†^ |
|  | Stage 2,3 | 17 (73.9) | 6 (26.1) |  |
| Type of surgery | Breast conserving surgery | 35 (79.5) | 9 (20.5) | 0.47^‡^ |
|  | Mastectomy | 9 (69.2) | 4 (30.8) |  |
| Time since surgery | Less than 45 days | 20 (80.0) | 5 (20.0) | 0.56^†^ |
|  | 45 days or more | 22 (73.3) | 8 (26.7) |  |
| Chemotherapy | Yes | 18 (78.3) | 5 (21.7) | 0.87^†^ |
|  | No | 26 (76.5) | 8 (23.5) |  |
| Hormone therapy | Antiestrogens (Tamoxifen) | 16 (88.9) | 2 (11.1) | 0.33^†^ |
|  | Aromatase inhibitors | 6 (66.7) | 3 (33.3) |  |
|  | No | 22 (73.3) | 8 (26.7) |  |
| Protocol of radiotherapy | Hypofractionated | 28 (77.8) | 8 (22.2) | 0.89^†^ |
|  | Conventionally fractionated | 16 (76.2) | 5 (23.8) |  |
| Comorbidity | Yes | 30 (78.9) | 8 (21.1) | 0.74^‡^ |
|  | No | 14 (73.7) | 5 (26.3) |  |
| Time required for hospital visit | Less than 30 minutes | 21 (75.0) | 7 (25.0) | 0.46^†^ |
|  | 30 minutes or more | 20 (83.3) | 4 (16.7) |  |
| Marital status | Married | 29 (70.7) | 12 (29.3) | 0.08^‡^ |
|  | Unmarried or other | 15 (93.8) | 1 (6.3) |  |
| Living arrangement | Living with family | 37 (75.5) | 12 (24.5) | 0.67^‡^ |
|  | Alone | 7 (87.5) | 1 (12.5) |  |
| Employment status | Working | 30 (81.1) | 7 (18.9) | 0.51^‡^ |
|  | Unemployed | 14 (70.0) | 6 (30.0) |  |

^†^χ^2^ test, ^‡^Fisher's exact test

Log LF/HF ≥ 0.301, the high log LF/HF group; log LF/HF < 0.301, the normal log LF/HF group.

Abbreviations: LF/HF, low frequency/high frequency
